# Items

**Published:** 1905-02

**Authors:** 


### ITEMS.

Apollinaris, that best of table waters, was awarded the Grand Prize, the highest award at the Saint Louis Exposition, for its high qualities as a natural mineral water. The United Agency Company, New York, is the distributing medium for the United States and Canada.

The Mellier Drug Company, of Saint Louis, has issued a calendar for 1905, which on the obverse presents a picture in color of Go-Shono an Apache medicine man. On the reverse the history of tongaline is given and the calendar proper is printed. This work of art will be sent to any physician on application to the publishers, 2112 Locust street, Saint Louis.

The Lambert Pharmacal Company, Saint Louis, has supplied the necessary month cards to renew the utility of the Listerine calendar for 1905. It is a useful office contrivance and may be obtained by those who have failed to receive it upon addressing the Lambert Pharmacal Company, Saint Louis.

The Antikamnia Chemical Company, Saint Louis, is distributing the antikamnia calendar for 1905, which is entitled Sympathy. It is a reproduction in color of Gatti's celebrated painting, which is considered a beautiful work of art. Upon the reverse is the calendar proper and several interesting items concerning Antikamnia tablets.

The Arlington Chemical Company, Yonkers, N. Y., with the beginning of the new year issued Volume II. of the Law and the Doctor. This pamphlet treats of the physician as a witness, and will be found a useful guide to any one who wishes to familiarise himself with court procedure on that important point, especially is it a handy reminder, containing, as it does, a digest of all the important questions likely to fall within the domain of the medical witness.

The Hudor Company, Buffalo, owing to an increased demand for pure drinking water, has enlarged its distilling plant and improved its methods, thus enabling it to offer pure distilled water at the reduced rate of 10 cents a gallon. This celebrated company also supplies Hudor lithia water, sarsaparilla, birch beer, lemon soda, ginger ale and other sparkling beverages at reasonable prices.

